# Prevalence of Electronic Cigarette Use Among Female Residents of Al-Ahsa, Kingdom of Saudi Arabia: A Cross-Sectional Study

**DOI:** 10.7759/cureus.66533

**Published:** 2024-08-09

**Authors:** Amnah A Alhuwayji, Abduallah M Alhamam, Mohammed Alramdan, Rahma Algadeeb

**Affiliations:** 1 Department of Preventive Medicine, Al-Ahsa Health Cluster, Ministry of Health, Al-Ahsa, SAU; 2 Department of Community Wellness, Al-Ahsa Health Cluster, Ministry of Health, Al-Ahsa, SAU

**Keywords:** vaping, smoking, saudi arabia, prevalence, electronic cigarettes

## Abstract

Background

Electronic cigarettes (e-cigarettes) have gained considerable popularity on a global scale, with an increasing prevalence among younger adults. The objective of this study was to investigate the prevalence, patterns, and determinants of e-cigarette use among women in Al-Ahsa, Saudi Arabia.

Methodology

A cross-sectional study was conducted between October 2023 and July 2024 involving 491 adult female participants. The data were collected using a structured questionnaire and subsequently analyzed using SPSS Version 26.0 (IBM Corp., Armonk, NY, USA). Descriptive and inferential statistics, including chi-square tests, were employed to assess relationships between e-cigarette use and various independent variables.

Results

The prevalence of e-cigarette use among participants was 17.5%. Significant factors associated with e-cigarette use included age (highest among women aged 21 to 30 years, p = 0.038), unemployment (p = 0.011), perceived poor health (p = 0.002), and having friends or family members who use e-cigarettes (p = 0.001). The primary reasons for using e-cigarettes were influence from friends (70.9%) and family members (54.7%), curiosity (33.7%), and appealing flavors (30.2%). A considerable proportion of users reported experiencing dependence and difficulty quitting.

Conclusions

The prevalence of e-cigarette use among female residents of Al-Ahsa is influenced by a complex interplay of social, demographic, and perceptual factors. The findings underscore the necessity for comprehensive interventions targeting social environments and educational initiatives and addressing misconceptions about the potential risks of e-cigarettes.

## Introduction

Cigarette smoking represents the most prevalent global public health concern [1]. The global prevalence of smoking is estimated at approximately 1.3 billion, with over 80% of smokers residing in low- and middle-income countries [2]. Furthermore, smoking prevalence rates among boys are consistently higher than those among girls, regardless of age or educational level [3].

Electronic cigarettes, referred to as e-cigarettes, have emerged as a prominent alternative to traditional tobacco smoking, rapidly gaining popularity across the globe [4]. This rise is reflected in the increasing number of users who view e-cigarettes as a less harmful option due to innovative technology that simulates smoking without the adverse effects associated with burning tobacco [5].

E-cigarettes are sophisticated devices engineered to deliver a smoking-like experience without the actual combustion of tobacco [6]. These devices consist of three main components: a battery, a heating element, and a liquid holder. The battery powers the heating element, which then heats the liquid contained in the holder, typically consisting of nicotine, flavorings, and other chemicals, transforming it into an aerosol that users inhale [7]. This technology not only replicates the physical act of smoking but also facilitates the hand-to-mouth ritual that many smokers find challenging to abandon [8].

The perception of e-cigarettes as a safer alternative to traditional tobacco smoking is largely based on the absence of tobacco combustion. The combustion of tobacco in traditional cigarettes produces smoke, which contains thousands of chemicals. Many of these chemicals are toxic and carcinogenic [9]. In contrast, e-cigarettes generate an aerosol, which is often misleadingly referred to as “vapor.” This aerosol is thought to contain fewer and lower levels of toxic substances than traditional cigarettes. This distinction has led many to view e-cigarettes as a less harmful option [10-12], potentially useful as a smoking cessation aid [13,14]. This may be true in the context of short-term exposure; however, this beneficial effect of reduced toxicant exposure may not be true following chronic or prolonged exposure to low levels of these harmful toxicants [15,16]. This has led to concerns being raised about the long-term health effects of e-cigarettes and has highlighted the need for further comprehensive studies to be conducted to gain a full understanding of their safety profile [17].

The use of e-cigarettes has significantly and steadily increased globally, reflecting a broad shift in smoking habits and the adoption of what many perceive as a safer alternative to traditional tobacco products [18]. According to a report by the World Health Organization, as of 2021, approximately 68 million people worldwide were identified as e-cigarette users, a notable rise from 58 million in 2018 [19]. This trend is projected to continue, with market analysts forecasting an increase in the global vaping population, driven by advancements in e-cigarette technology, increasing market penetration, and an expanding variety of flavor options appealing to a broader consumer base [20].

Demographic variations in e-cigarette use reveal significant disparities between different age groups and genders. These disparities provide insights into targeted marketing strategies and cultural influences that impact the adoption of vaping [21]. Studies consistently show that e-cigarette use is more prevalent among younger adults and teenagers, who are drawn to the novel aspects of vaping, such as flavor variety and the perceived modernity of e-cigarettes compared to traditional smoking. In the United States, the prevalence of ever having used an electronic nicotine product was higher among young adults (aged 18-24) than among older adults. Almost a third of young adults had used an electronic nicotine product compared with about a quarter of adults aged 25-34, which is much higher than among adults aged 65 and over (4.1%) [22-24]. However, in some regions, such as Eastern Europe and Asia, the gender gap in e-cigarette use is less pronounced, suggesting that regional cultural factors also play a critical role in influencing e-cigarette consumption patterns [25]. These demographic insights are crucial for public health officials and policymakers aiming to devise effective strategies to address the rising trend of e-cigarette use and mitigate its health impacts [26].

E-cigarettes emit aerosols containing compounds that can be potentially toxic. The main components in e-cigarette aerosols are propylene glycol, glycerol, and hazardous metals such as lead and cadmium. These substances can potentially affect DNA, reducing its ability to repair itself during replication [27]. In addition, nicotine has been shown to be harmful, particularly in young people and pregnant women [28]. However, some researchers argue that vaping is relatively less harmful than smoking, which has been conclusively linked to several major health problems such as lung cancer, chronic obstructive pulmonary disease, and interstitial lung disease [29].

In Saudi Arabia, a recent study found that about a quarter of study participants had tried e-cigarettes at least once in their lifetime, and e-cigarette use was significantly higher among young adult males with higher levels of education [30]. Another study conducted among Saudi students found that 22.8% and 15.6% used them for recreational and smoking cessation purposes, respectively [31]. The significance of e-cigarettes goes beyond mere prevalence; it presents unique challenges and opportunities for public health policy, especially given the diverse cultural and regulatory landscapes within the Kingdom [32,33].

In addition, the legal status of e-cigarettes has evolved in parallel with an increased understanding of their potential health impacts. The Saudi Food and Drug Authority regulates e-cigarettes similar to other tobacco products, requiring clear health warnings on packaging about the dangers of nicotine addiction [34]. Furthermore, the sale of e-cigarettes is prohibited in areas where traditional tobacco smoking is prohibited, reflecting a cautious approach to public health policy. These regulations are part of a broader effort to mitigate the health risks associated with nicotine products, particularly among youth and non-smokers who might view e-cigarettes as a harmless alternative to smoking [35].

The necessity for research on e-cigarette use among women in Saudi Arabia is not solely driven by concerns regarding health-related effects. It is also shaped by several distinctive cultural and social factors that are specific to this population [36]. Culturally, the behaviors and health practices of women in Saudi Arabia are often less documented due to traditional gender roles and societal expectations, which can differ significantly from those of their male counterparts [37]. Socially, environments present a distinct set of influences, including peer interactions and exposure to new social norms that may affect health behaviors such as vaping [38]. This study aims to address a significant gap in gender-specific research on vaping by focusing on young Saudi women, a group that has historically been underrepresented in tobacco-related studies. By examining the prevalence, patterns, and determinants of e-cigarette use specifically among adult women, this research seeks to uncover insights that are critical for developing targeted health interventions. It is, therefore, imperative that any interventions developed address not only the general risks associated with e-cigarette use but also the ways in which these risks impact adult females, who may experience different social pressures and health effects compared to their male peers.

## Materials and methods

Study design

This study employed an analytic cross-sectional design to determine the prevalence and explore the determinants of e-cigarette use among female residents in Al-Ahsa between October 2023 and July 2024. The selected design was particularly well-suited to estimating the prevalence and assessing the relationships between e-cigarette use and potential predictor variables within a defined population at a specific point in time. This approach allowed for a comprehensive snapshot analysis of behavioral health patterns within the community.

Study setting

The research was conducted in the region of Al-Ahsa, Saudi Arabia, a culturally and demographically diverse area. Al-Ahsa was selected for its considerable population size and diversity, which provided a comprehensive context for understanding health behaviors across various community subgroups.

Sample size calculation

The sample size was estimated with an online sample size calculator (OpenEpi tool) using a margin of error of 5%, a confidence interval of 95%, a response distribution of 50%, and depending on an average adult female population of nearly 275,000 in the Al-Ahsa region according to Saudi Census 2022 [39]. The required sample size was estimated to be 384 participants.

Sampling technique

Participants were recruited using a simple random sampling technique. Various residential areas within Al-Ahsa were identified based on demographic and geographic characteristics, focusing on the most popular places in Al-Hofuf, Al-Mbarraz, and rural or remote areas.

Inclusion criteria

Female residents of Al-Ahsa, aged 18 years and above, were invited to participate in the study. This demographic focus was essential, as it aimed to capture a wide range of experiences and behaviors related to e-cigarette use.

Exclusion criteria

Individuals who were not residents of Al-Ahsa and those who were unable to provide consent due to cognitive impairment or language barriers were excluded from the study. This approach helped maintain the study’s focus on a specific population group and ensured that all participants could actively and knowingly participate.

Data collection tools

The data were collected through a structured questionnaire that was initially developed in English [40] and then translated into Arabic, the primary language of the participants. This translation process was undertaken to ensure clarity and cultural relevance. The translation process included a back-translation method to validate the accuracy of the translation. Bilingual interviewers, trained in the administration of the questionnaires, ensured the reliability of the responses by allowing participants to clarify any doubts directly. This approach also facilitated the accurate collection of data.

Data analysis

SPSS Statistics version 26.0 (IBM Corp., Armonk, NY, USA) was employed for statistical analysis. Descriptive statistics were utilized to summarize the data, providing an overview of sociodemographic details and e-cigarette usage patterns. Inferential statistics, including chi-square tests, were employed to examine the relationships between e-cigarette use and various independent variables, such as age, socioeconomic status, and educational level. A significance threshold of p < 0.05 was used to determine statistical significance.

Ethical considerations

The study was conducted in accordance with the ethical guidelines stipulated by the local health authority. Ethical approval was obtained from the Ethics Committee of King Faisal University (approval number: KFU-REC-2023-DEC-ETHICS1703), ensuring that all procedures performed were in accordance with ethical standards. The confidentiality and anonymity of participants were strictly maintained. Each participant was required to provide written informed consent, thereby ensuring ethical compliance and participant awareness of the study’s nature and purpose. Furthermore, participants were informed of their right to withdraw from the study at any time without consequence.

## Results

Most participants were in the 21-30-year age group (43.0%), with the majority being Saudi nationals (98.0%). Educational attainment was notably high, with almost half having a university education (49.5%). The majority were single (61.3%) and predominantly non-medical students or employees (74.1%). Concerning smoking habits, 79% of participants had never smoked, while 11.4% were current smokers. Among smokers, e-cigarette use was the most common form of smoking (33%), followed by dual usage of electronic and traditional cigarettes (29.1%). In terms of the duration of smoking, a significant number had been smoking for five years or more (33%). The majority of participants (66.0%) rated their current health condition as good (Table 1).

**Table 1:** Demographic characteristics and smoking habits (N = 491).

Variable	Number	%
Age, year		
18–20	121	24.6%
21–30	211	43.0%
31–40	97	19.8%
>40	62	12.6%
Nationality		
Saudi	481	98.0%
Non-Saudi	10	2.0%
Educational level		
Below secondary education	17	3.5%
Secondary education	210	42.8%
University education	243	49.5%
Postgraduate	21	4.3%
Marital status		
Single	301	61.3%
Married	166	33.8%
Divorced/widow	24	4.9%
Work		
Not working	93	18.9%
Medical student/Employee	34	6.9%
Non-medical student/Employee	364	74.1%
Smoking		
Never smoked	388	79.0%
Ex-smoker	47	9.6%
Current smoker	56	11.4%
What kind do you smoke? (n = 103)		
Electronic cigarettes	34	33.0%
Traditional cigarettes	9	8.7%
Shisha	8	7.8%
Electronic and traditional cigarettes	30	29.1%
Electronic cigarettes and shisha	22	21.4%
Duration of smoking, year (n = 103)		
<1	23	25.3%
1–2	24	26.4%
3–4	14	15.4%
≥5	30	33.0%
What do you think about your current health condition?		
Poor	12	2.4%
Average	155	31.6%
Good	324	66.0%

The majority (88.6%) of the participants were aware of e-cigarettes, yet only a smaller fraction (14.7%) had experienced a strong urge to vape. Social influences had a significant impact on the prevalence of e-cigarette use. A substantial proportion of respondents reported that friends (26.5%) or family members (brothers 23.2%, fathers 7.1%) used e-cigarettes. Concerning the health risks associated with e-cigarettes, the majority of respondents identified risks to lung and cardiovascular functions (65.1%). Nevertheless, there was a notable lack of awareness regarding other potential risks, such as nicotine addiction (22.1%) and negative nutritional impacts (12.8%). The primary sources of information about e-cigarettes were friends (61.5%) (Table 2).

**Table 2:** Knowledge and awareness about electronic cigarettes (N = 491).

Variable	Number	%
Have you heard of using electronic cigarettes if you are not a smoker?		
Yes	435	88.6%
No	56	11.4%
Have you ever felt a strong urge to vape?		
Yes	72	14.7%
No	419	85.3%
Has someone close to you been an electronic cigarette user in their life?		
None	198	40.3%
A friend	130	26.5%
Brother	114	23.2%
Father	35	7.1%
Both parents	10	2.0%
Mother	4	0.8%
What are the health risks associated with electronic cigarettes? (n = 86)		
Risks to lung and cardiovascular functions	56	65.1%
Relationship with nutritional intake due to decreased appetite	11	12.8%
Risk of nicotine addiction	19	22.1%
Source of information about electronic cigarettes		
Friends	302	61.5%
Cafes	163	33.2%
Newspaper	109	22.2%
Work	58	11.8%
Poster	56	11.4%

A minority of the study participants (17.50%) reported having used e-cigarettes. Among these users, the frequency of vaping varied, with the majority (56.98%) vaping one to four times daily. Notably, about a third of the e-cigarette users (33.72%) started vaping within the first five minutes of waking up. The primary sources for obtaining e-cigarettes were e-cigarette shops (60.47%) and friends (34.88%). Usage locations varied widely, with most users vaping in cars (81.40%) and homes (70.93%). Social contexts of vaping also varied, with a majority smoking with friends (62.79%) or alone (54.65%). Regarding product preferences, a variety of liquid flavors was the most valued (72.09%) (Table 3).

**Table 3:** Electronic cigarette smoking, frequency, pattern, and source (N = 491).

Electronic cigarette smoking	Number	%
Do you use or have you ever used electronic cigarettes?		
Yes	86	17.50%
No	405	82.50%
Frequency of vaping (n = 86)		
1–4	49	56.98%
5–14	12	13.95%
15–19	7	8.14%
20–29	7	8.14%
≥30	11	12.79%
On days when you can freely use your electronic cigarette, as soon as you wake up from sleep, when do you use an electronic cigarette for the first time? (n = 86)		
<5 minutes	29	33.72%
6–15 minutes	9	10.47%
16–30 minutes	24	27.91%
31–60 minutes	8	9.30%
61–120 minutes	7	8.14%
>120 minutes	9	10.47%
Source of having electronic cigarettes (n = 86)		
Electronic cigarette shop	52	60.47%
Friends	30	34.88%
Market stall	9	10.47%
Supermarket	8	9.30%
Internet	8	9.30%
Pharmacy	3	3.49%
Family	3	3.49%
Where do you vape? (n = 86)		
Car	70	81.40%
Home	61	70.93%
cafe	28	32.56%
Work	24	27.91%
Parties	23	26.74%
School	8	9.30%
With whom do you smoke? (n = 86)		
Alone	47	54.65%
With friends	54	62.79%
With family	7	8.14%
All of them	20	23.26%
Which of the following characteristics of an electronic cigarette are important to you? (n = 86)		
A variety of liquid flavors	62	72.09%
Fast battery charging	30	34.88%
Long battery life	28	32.56%
Provides good steam quality	26	30.23%
In the shape of a cigarette	25	29.07%

**Figure 1:**
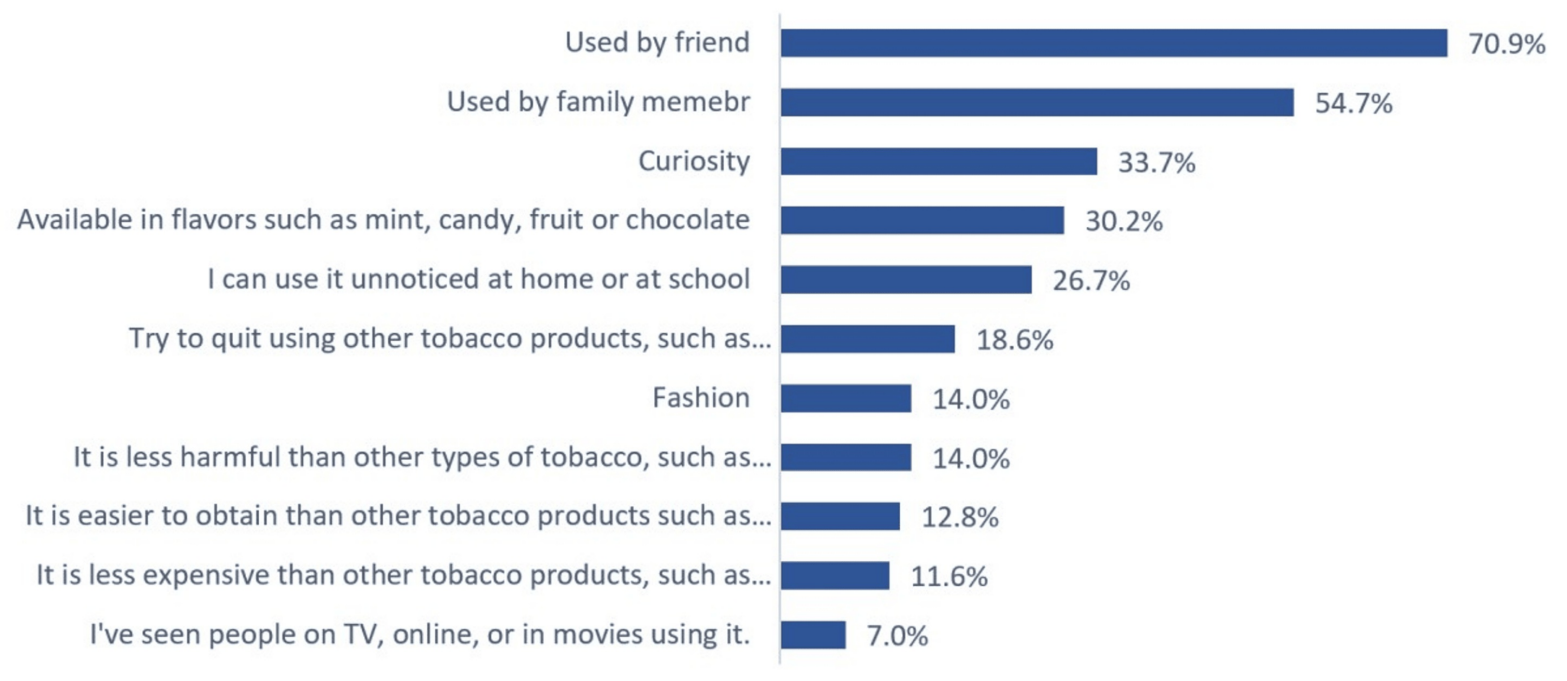
Reasons for using electronic cigarettes among users (N = 86).

The most prevalent reason why study participants use e-cigarettes, as reported by 70.9% of the users, was influence from friends. Following closely, 54.7% of participants were influenced by family members using e-cigarettes. Curiosity about e-cigarettes motivated 33.7% of the users. Other reasons for the popularity of e-cigarettes included their availability in appealing flavors such as mint, candy, or chocolate, which attracted 30.2% of users. Additionally, less than one-third (26.7%) of users appreciated the discreetness of e-cigarettes, which allowed them to vape unnoticed at home or school. Fewer participants used e-cigarettes as a cessation tool for other tobacco products (18.6%), for fashion (14%), or because they perceived them as less harmful than traditional cigarettes (14%). Moreover, 12.8% found e-cigarettes easier to obtain than other tobacco products, while 11.6% considered them less expensive. A small fraction (7%) mentioned seeing e-cigarettes used by people on TV, online, or in movies (Figure 1).

A substantial proportion, 25.6%, reported waking up at night to use their e-cigarette, with over 72% doing so multiple nights per week. Moreover, 38.4% of respondents believed it is challenging to quit using e-cigarettes, and 39.5% found it difficult to abstain from vaping in prohibited areas. Feelings of distress when unable to use an e-cigarette were reported by 37.2% of the participants, and 40.7% experienced stress, insomnia, or anxiety when they could not vape. Despite these challenges, a majority (61.6%) believed that e-cigarettes can aid in quitting smoking, and 45.3% perceived a social benefit to e-cigarette use, believing that it is associated with having more friends. Interestingly, opinions on the safety and addictiveness of e-cigarettes compared to traditional cigarettes were mixed, with a slight majority viewing them as safer but opinions on their addictiveness were divided (Table 4).

**Table 4:** Attitude and perception about electronic cigarettes among users (N = 86).

Attitude and perception of electronic cigarettes	Number	%
Do you sometimes wake up at night to use your electronic cigarette?		
Yes	22	25.6%
No	64	74.4%
If yes, how many nights		
1–2	6	27.3%
3	8	36.4%
≥4	8	36.4%
Are you using an electronic cigarette now because it is really hard to quit?		
Yes	33	38.4%
No	53	61.6%
Is it difficult to refrain from using an electronic cigarette in places where you are not supposed to?		
Yes	34	39.5%
No	52	60.5%
When you haven’t used an electronic cigarette for a while or when you’ve tried to stop using one), did you feel more upset because you couldn’t use an electronic cigarette?		
Yes	32	37.2%
No	54	62.8%
Have you felt stressed, insomnia, or anxious because you cannot smoke a cigarette?		
Yes	35	40.7%
No	51	59.3%
Do you think young people who use electronic cigarettes have more friends?		
Yes	39	45.3%
No	47	54.7%
Do you think that electronic cigarettes are safer than regular cigarettes?		
Yes	37	43.0%
No	49	57.0%
Do you think smoking electronic cigarettes can help you quit smoking cigarettes?		
Yes	53	61.6%
No	33	38.4%
Compared to cigarettes, electronic cigarettes are		
Less addictive	27	31.4%
More addictive	19	22.1%
Same hazard	27	31.4%
Do not know	13	15.1%

A significant correlation was observed between age and e-cigarette usage (p = 0.038). The highest usage was observed in the 21-30-year age group, with 21.30% of individuals in this age group reporting e-cigarette use. Employment status and perceptions of health also showed significant associations with e-cigarette use. Those not employed exhibited a higher usage rate (23.40%, p = 0.011), and those who considered their health to be poor were more likely to use e-cigarettes (53.85%, p = 0.002). Those who experienced a strong urge to vape were significantly more likely to be current vape users (p = 0.001). Another key finding is the strong influence of peer and familial connections on e-cigarette use. Individuals with a friend or family member who used e-cigarettes were significantly more likely to use them themselves (p = 0.001). The source from which participants received information about electronic cigarettes had a significant impact on their likelihood of vaping. In particular, information from friends appeared to exert a greater influence than information from other sources (p = 0.001) (Table 5).

**Table 5:** Factors associated with electronic cigarette use (N = 491). *: Significant at p < 0.05.

Factor	Do you use or have you used electronic cigarettes?				P-value
	Yes (n = 86)		No (n = 406)		
	Number	%	Number	%	
Age, year					0.038*
18–20	14	11.60%	107	88.40%	
21–30	45	21.30%	166	78.70%	
31–40	20	20.60%	77	79.40%	
>40	7	11.11%	56	88.89%	
Nationality					0.284
Saudi	82	17.00%	399	83.00%	
Non-Saudi	4	40.00%	6	60.00%	
Educational level					0.203
Below secondary education	5	29.40%	12	70.60%	
Secondary education	29	13.80%	181	86.20%	
University education	47	19.26%	197	80.74%	
Postgraduate	5	23.80%	16	76.20%	
Marital status					0.27
Single	47	15.56%	255	84.44%	
Married	33	19.90%	133	80.10%	
Divorced/Widow	6	25.00%	18	75.00%	
Work					0.011*
Not working	22	23.40%	72	76.59%	
Medical student/Employee	0	0.00%	34	100.00%	
Non-medical student/Employee	64	17.60%	300	82.40%	
What do you think about your current health condition?					0.002*
Poor	7	53.85%	6	46.15%	
Average	33	21.30%	122	78.70%	
Good	46	14.20%	278	85.80%	
Have you ever felt a strong urge to vape?					0.001*
Yes	48	65.75%	25	34.25%	
No	38	9.10%	381	90.90%	
Has someone close to you been an electronic cigarette user in their life?					0.001*
None	8	4.00%	190	96.00%	
A friend	49	37.40%	82	62.59%	
Siblings	18	15.80%	96	84.20%	
Father	5	14.30%	30	85.70%	
Mother	2	50.00%	2	50.00%	
Both parents	4	40.00%	6	60.00%	
Source of information about electronic cigarettes					0.001*
Friends	76	25.08%	227	74.92%	
Cafes	15	9.20%	148	90.80%	
Newspaper	2	1.80%	107	98.20%	
Poster	3	5.40%	53	94.60%	
Work	11	19.00%	47	81.00%	

## Discussion

This cross-sectional study offers valuable insights into the prevalence, patterns, and determinants of e-cigarette use among women in Al-Ahsa, Saudi Arabia. The findings reveal a multifaceted picture, indicating the influence of social, demographic, and perceptual factors on e-cigarette usage within this population.

The overall prevalence of e-cigarette use among the study participants was found to be 17.5%, a notable figure that warrants attention from public health authorities. This prevalence aligns with global trends indicating a rising popularity of e-cigarettes, particularly among younger adults [41-43]. However, it is essential to contextualize this finding within the cultural and social norms of Saudi Arabia, where traditional tobacco smoking among women has historically been less prevalent compared to other regions [44].

One of the most striking findings is the strong influence of social factors on e-cigarette use. The study revealed that having friends or family members who use e-cigarettes significantly increased the likelihood of participants using e-cigarettes themselves. This observation is consistent with existing literature highlighting the role of peer influence and social modeling in shaping health behaviors, particularly among young adults [45]. The social context of vaping, with a majority of users reporting smoking alone or with friends, further reinforces the interplay between e-cigarette use and social dynamics, as evidenced by previous studies [46,47].

The age distribution of e-cigarette users in this study revealed a peak in usage among the 21-30-year age group, which corroborates previous research indicating a higher prevalence of e-cigarette use among younger populations [30]. This trend may be attributed to several factors, including the appeal of novel technologies, targeted marketing strategies, and the perceived modernity associated with e-cigarettes among younger adults [48].

It is noteworthy that employment status emerged as a significant factor associated with e-cigarette use, with higher usage observed among those not working. This finding may be linked to various factors, including increased leisure time, fewer financial constraints, or different social environments and stressors experienced by unemployed individuals [49]. Further investigation of the relationship between employment status and e-cigarette use could provide valuable insights for the development of targeted interventions [50].

The study also highlighted the relationship between perceived health status and e-cigarette use. Participants who considered their health to be poor were more likely to use e-cigarettes. This observation aligns with the potential use of e-cigarettes as a perceived “healthier” alternative to traditional smoking among individuals with existing health concerns [4,51]. However, it is important to be cautious as the safety of e-cigarettes is still debated, with emerging research indicating the presence of potentially harmful substances in e-cigarette aerosols and possible long-term health risks [52].

The reasons cited by participants for using e-cigarettes offer valuable insights into the motivations driving this behavior. The influence of friends and family members emerged as the most prominent factor, further underscoring the social dimensions of e-cigarette use. Curiosity and the availability of appealing flavors also played significant roles, reflecting the allure of novelty and sensory experiences associated with vaping. Notably, most e-cigarette users believed that e-cigarettes can help quit smoking, suggesting that potential misconceptions about the efficacy of e-cigarettes for smoking cessation may exist within this population [53,54].

The study also illuminated the attitudes and perceptions of female e-cigarette users regarding their habits and beliefs about vaping. The reported instances of waking up at night to use e-cigarettes, difficulty refraining from vaping in prohibited areas, and feelings of distress or anxiety when unable to vape, point toward potential psychological and physical dependencies [55,56]. These findings raise concerns about the addictive nature of e-cigarettes and the necessity for targeted interventions to address dependence and promote cessation.

While the majority of participants believed that e-cigarettes could assist in smoking cessation, opinions on the safety and addictiveness of e-cigarettes in comparison to traditional cigarettes were divided. These findings underscore the necessity for educational campaigns and health literacy initiatives to address misconceptions and provide accurate information on the potential benefits and risks surrounding e-cigarettes as a cessation tool [57].

The study’s findings have significant implications for public health policies and interventions aimed at addressing e-cigarette use among young women in Saudi Arabia. The influence of social factors underscores the importance of developing comprehensive strategies that target not only individual behaviors but also the social environments and peer networks that shape these behaviors. Peer-led education programs, community-based interventions, and social media campaigns could be effective in addressing the social dimensions of e-cigarette use [58].

Furthermore, the observed age-related patterns in e-cigarette use emphasize the necessity for targeted prevention and awareness programs tailored to younger populations. Educational initiatives within universities and schools could play a pivotal role in disseminating accurate information about the potential benefits and risks of e-cigarettes, countering misconceptions and misinformation [59].

It is also crucial to examine the broader societal factors that may contribute to e-cigarette use, such as employment status and perceived health status. Interventions that address socioeconomic determinants of health, provide support for unemployed individuals, and promote overall well-being may be effective in mitigating the factors associated with increased e-cigarette use [60].

Limitations

The cross-sectional design of the study precludes the establishment of causal relationships between the factors examined and e-cigarette use. Additionally, the reliance on self-reported data may introduce potential biases or underreporting. The study was conducted in a specific geographic location (Al-Ahsa, Saudi Arabia), which limits the generalizability of the findings to other regions or populations. Furthermore, the study did not include biological markers or objective measures of e-cigarette use, relying solely on self-reported data. Finally, the study did not explore the long-term health impacts or potential adverse effects of e-cigarette use among the participants.

## Conclusions

This study provides valuable insights into the prevalence, patterns, and determinants of e-cigarette use among female residents of Al-Ahsa, Saudi Arabia. The findings indicate that social factors, age, employment status, and health perceptions significantly influence e-cigarette usage within this population. These insights underscore the need for multifaceted public health interventions that address not only individual behaviors but also the broader societal and environmental factors shaping e-cigarette use. By addressing the prevailing misconceptions surrounding vaping, promoting health literacy, and targeting the social dimensions of vaping, policymakers and health authorities can develop more effective strategies to mitigate potential risks and promote healthier behaviors among young women in Saudi Arabia.
